# Reliability of the L3 Transverse Process as a Landmark for Lumbar Spine Enumeration Across Different Vertebral Segmentation Patterns: A Retrospective CT Study

**DOI:** 10.3390/diagnostics16203409

**Published:** 2026-10-20

**Authors:** Jakub Jankowski, Natalia Maślanek, Marcin Glin, Marcin Jasiewicz, Agnieszka Pawełczyk, Michał Polguj, Maciej Radek

**Affiliations:** 1 Department of Neurosurgery, Spine and Peripheral Nerve Surgery, Medical University of Lodz, Żeromskiego 113, 90-549 Lodz, Poland; natalia.maslanek@stud.umed.lodz.pl (N.M.); marcin.glin@stud.umed.lodz.pl (M.G.); agnieszka.pawelczyk@umed.lodz.pl (A.P.); maciej.radek@umed.lodz.pl (M.R.); 2 Department of Medical Imaging Technology, Faculty of Medicine, Medical University of Lodz, Narutowicza 60, 90-136 Lodz, Poland; marcin.jasiewicz@umed.lodz.pl; 3 Department of Normal and Clinical Anatomy, Chair of Anatomy and Histology, Medical University of Lodz, 90-752 Lodz, Poland; michal.polguj@umed.lodz.pl

**Keywords:** anatomical landmark, computed tomography, lumbar spine, lumbosacral transitional vertebrae, morphology, spinal segmentation, transverse process, vertebral numbering

## Abstract

**Background/Objectives**: Vertebral numbering on limited lumbosacral imaging is difficult when the thoracic or lumbar count varies. The L3 transverse process has been proposed as a landmark because it is often the longest, but this has not been tested across segmentation patterns. This study assessed whether L3 as the level bearing the longest transverse process is independent of thoracic and lumbar vertebral count on whole-spine CT. **Methods**: Of 499 trauma CT examinations with confirmed numbering, all 49 non-standard thoracolumbar formulas were included, together with 17 C7T12L5 spines that remained after exclusion of transitional and numerical anomalies at every level (*n* = 66). Bilateral lumbar transverse-process lengths were measured on multiplanar reconstructions. The longest level was identified by morphological and presacral numbering. Associations with thoracic patterns (C7T11, *n* = 10; C7T12, *n* = 43; C7T13, *n* = 13) were tested with the Fisher–Freeman–Halton exact test and Monte Carlo simulation. Nine primary analyses were adjusted with the Holm procedure. Inter-observer agreement was assessed with an intraclass correlation coefficient. **Results**: In this enriched cohort, L3 was the longest level in 45/66 cases (68.2%) based on the single longest transverse process and 49/66 (74.2%) based on bilateral mean length. These percentages are not population prevalences. Within the C7T12 group, the corresponding proportions were 76.7% and 83.7%, respectively. By bilateral mean length, L3 was longest in 30.0% of C7T11, 83.7% of C7T12 and 76.9% of C7T13 cases (exact *p* = 0.003, Holm-adjusted *p* = 0.014). The single-process association did not remain significant after Holm correction (adjusted *p* = 0.058). The 22nd presacral vertebra showed a comparable dependence on thoracic pattern (Holm-adjusted *p* = 0.041 and 0.001 for the single-process and bilateral-mean analyses, respectively). Inter-observer ICC was 0.88 for individual transverse-process lengths and 0.93 for bilateral means. **Conclusions**: Neither L3 nor the 22nd presacral vertebra is a segmentation-independent marker of lumbar identity. Full-spine counting remains necessary when precise numbering is required.

## 1. Introduction

The reference standard for vertebral enumeration is craniocaudal counting on whole-spine imaging, typically beginning at C2 or T1 [1,2,3,4]. However, routine lumbar CT and MRI examinations frequently have a restricted field of view that does not permit direct counting from the cervical spine [1,5,6]. This restricted field of view makes correct identification of vertebral segmentation difficult, particularly in the presence of anatomical variants such as numerical variation in rib-bearing thoracic segments or lumbar vertebral count or lumbosacral transitional vertebrae (LSTV) [1,2,7,8,9]. LSTV can be morphologically characterized using established classification systems such as the Castellvi classification; however, morphological classification alone does not establish the absolute numerical identity of the transitional segment [1,2]. Moreover, numerical and transitional variants may involve more than one region of the vertebral column, and thoracolumbar transitional anatomy has been associated with both abnormal rib counts and lumbosacral transitional anatomy [7,10,11,12]. Wrong-level interventions remain a recognized risk in spinal surgery and interventional pain procedures, with potentially serious clinical and medicolegal consequences [1,2,13].

Numerous spinal and paraspinal markers have been proposed to aid vertebral enumeration on limited imaging, including the iliac crest line, iliolumbar ligament, aortic bifurcation, right renal artery, conus medullaris, and various neural or morphological features [1,5,6,14,15,16,17,18]. Nevertheless, their reliability is limited, particularly in the presence of transitional or numerical vertebral variants [1,5,6,8,16,17,18]. The iliolumbar ligament, for example, was initially proposed as a reliable marker of the L5 vertebra [15], whereas subsequent studies demonstrated substantial variability in its vertebral origin in patients with LSTV and other segmentation anomalies [8,16,17]. Similarly, vascular, neural, and skeletal landmarks may vary in their relationship to conventionally numbered vertebral levels and therefore cannot consistently replace direct whole-spine enumeration [1,5,6,14,17,18]. Consequently, the traditional approach of counting caudally from C2 or T1 on a full-spine localizer remains the most accurate method, yet it is often unavailable in routine lumbosacral examinations [1,2,3,4].

Recent reports on the lengths of the lumbar transverse processes have suggested the possibility of a simple, reproducible bony landmark. In particular, Rädel and Paton reported in an osteological study of dried human spinal columns that L3 most frequently demonstrated the longest mean transverse process length in both LSTV-free controls and specimens with LSTV, and proposed this feature as a potential aid to lumbar spine enumeration [19]. However, the usefulness of a level-specific anatomical landmark depends not only on how frequently it occurs within a given cohort, but also on whether its relationship to vertebral identity remains stable across numerical variants of the spine. From a developmental perspective, numerical changes at different vertebral boundaries may coexist and have been interpreted as homeotic shifts in regional vertebral identity [20]. This provides a biological rationale for investigating whether transverse process morphology and the level bearing the longest transverse process remain constant across different vertebral formulas. The influence of variation in thoracic and lumbar vertebral counts on the level bearing the longest transverse process has not been systematically evaluated. The aim of the present study was therefore to assess whether L3 as the level bearing the longest lumbar transverse process represents a segmentation-independent anatomical marker by evaluating its relationship with different vertebral segmentation patterns using whole-spine CT as the anatomical reference.

## 2. Materials and Methods

### 2.1. Study Design and Ethics

This retrospective, observational study was approved by the Bioethics Committee of the Medical University of Lodz (approval number: RNN/09/25/KE). All procedures were conducted in accordance with the Declaration of Helsinki and relevant institutional guidelines. Because the study involved the retrospective analysis of anonymized clinical CT data, the requirement for individual informed consent was waived by the Bioethics Committee.

### 2.2. Study Population and Data Acquisition

The dataset was derived from a review of whole-body trauma CT examinations performed in polytrauma patients admitted to the emergency department of a tertiary referral hospital in Poland between January 2022 and December 2024. All examinations were acquired using a 16-detector-row multidetector CT scanner (Optima 540 (GE Healthcare, Waukesha, WI, USA)) according to the institutional whole-body trauma protocol. The patients were scanned in the supine position with a head-first orientation. The scan range included the cervical spine followed by continuous helical acquisition of the thorax and abdomen.

Imaging was performed with a gantry rotation time of 0.8 s and a tube voltage of 120 kV. Automated tube current modulation was applied, with a maximum tube current of 430 mA adjusted according to patient attenuation. Image reconstruction was performed with a slice thickness of 1.25 mm and a reconstruction interval equal to the slice thickness, allowing multiplanar and three-dimensional reconstructions.

When patients had undergone multiple trauma scans during the study period, only the most recent CT examination was included. Cases with incomplete visualization of the thoracolumbar junction or poor image quality precluding reliable measurement were excluded.

### 2.3. Image Analysis and Reviewers

All CT examinations were initially evaluated by two final-year medical students who had completed the Radiology course as part of their medical degree. The assessments were subsequently reviewed by a board-certified neurosurgeon with subspecialty training in spinal disorders, interventional pain management, and neuroradiology, who served as the senior reviewer for quality control. In cases of diagnostic uncertainty regarding vertebral numbering, transitional anatomy, or measurement, the final decision was reached by consensus after joint review.

As part of the reliability analysis, all 66 studies were re-evaluated independently by a neurosurgeon using the same measurement protocol and without access to the original measurement results. Inter-observer reliability regarding the length of the transverse processes was assessed by comparing the results obtained from this independent evaluation with the original measurements.

Image analysis was performed using RadiAnt DICOM Viewer (Medixant, Poznań, Poland), version 2025.2 with multiplanar reconstructions (MPR) and three-dimensional rendering.

### 2.4. Vertebral Segmentation and Group Allocation

**Figure 1:**
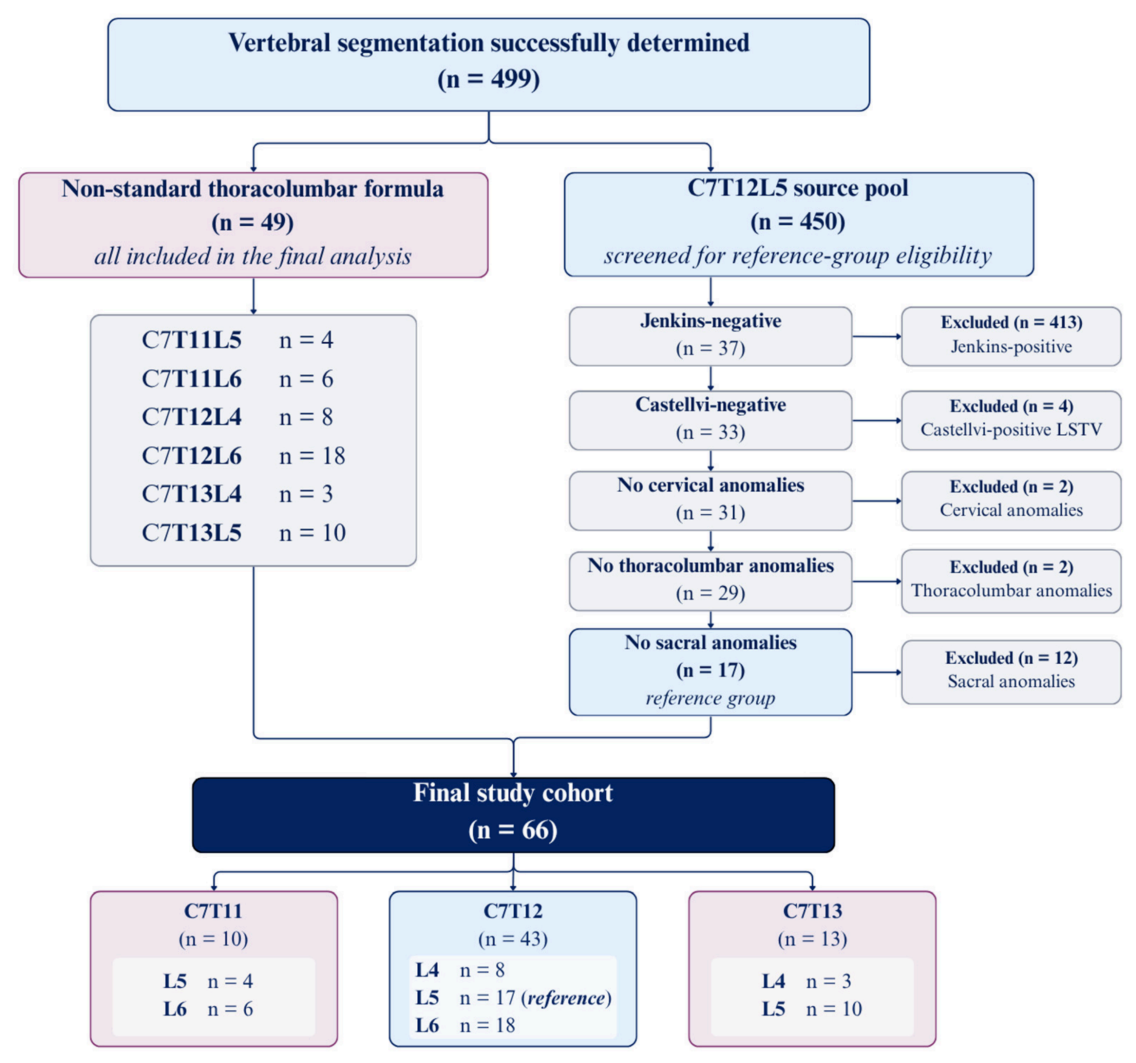
Flow diagram of patient selection and allocation to the final study groups.

Vertebral segmentation was successfully determined in 499 patients. Forty-nine had a non-standard thoracolumbar formula and were included in full: C7T11L5 (*n* = 4), C7T11L6 (*n* = 6), C7T12L4 (*n* = 8), C7T12L6 (*n* = 18), C7T13L4 (*n* = 3) and C7T13L5 (*n* = 10). The remaining 450 patients had the numerical formula C7T12L5. From this group, the reference sample was restricted to spines with no transitional or numerical anomaly at any level. The Jenkins classification, a recently proposed system [21], was applied as an additional exclusion filter rather than as a prevalence instrument: 37 patients did not meet Jenkins criteria. Exclusion of Castellvi-positive lumbosacral transitional vertebrae [22] left 33; exclusion of cervical anomalies left 31; exclusion of thoracolumbar anomalies left 29; and exclusion of sacral anomalies left 17. These 17 examinations are therefore the complete set of source spines that remained anatomically typical after every applied filter, not a size-capped sample of C7T12L5 studies. The patient-selection process and allocation to the final study groups are summarized in Figure 1.

For the primary analysis, patients were stratified into three main groups according to thoracic segmentation pattern (i.e., number of rib-bearing thoracic vertebrae): C7T11 (*n* = 10), C7T12 (*n* = 43), and C7T13 (*n* = 13). This stratification was used to evaluate whether the anatomical level bearing the longest lumbar transverse process differed according to thoracic vertebral count. Because the C7T12 group contained patients with four, five, and six lumbar vertebrae, additional analyses were performed to examine the potential influence of lumbar vertebral count on the observed associations. Accordingly, secondary analyses stratified patients according to lumbar vertebral count as L4 (*n* = 11), L5 (*n* = 31), and L6 (*n* = 24). In addition, a sensitivity analysis compared patients with standard C7T12L5 segmentation (*n* = 17) with the pooled group of patients with non-standard vertebral configurations (*n* = 49). The individual thoracolumbar configurations were also retained for descriptive analyses to avoid obscuring variation within the three primary thoracic groups.

### 2.5. Measurement of Transverse Processes and Iliac Crest Line

**Figure 2:**
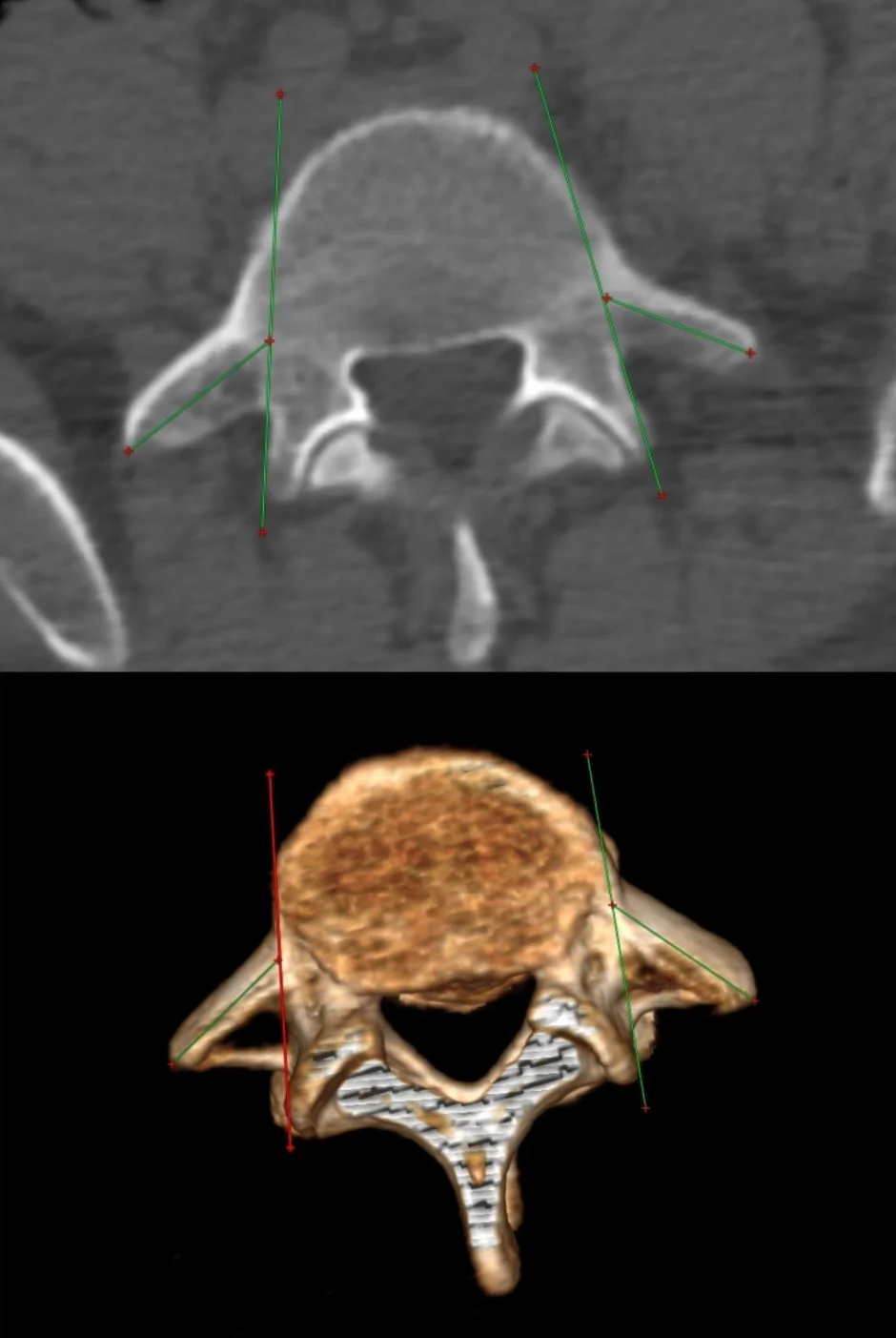
Measurement protocol for lumbar transverse process length on multiplanar CT reconstructions and 3D reconstruction (example image showing the longest distance from the pedicle-lamina junction to the tip of the transverse process).

The lengths of the transverse processes of all lumbar vertebrae were measured bilaterally on multiplanar reconstructions. The measurements were performed according to a standardized methodology as described by Rädel et al. [19]. The measurements were based on the longest distance from the junction of the transverse process with the pedicle/lamina to the tip of the process (Figure 2). Left- and right-sided measurements were recorded separately. For each examination, the vertebral level bearing the single longest transverse process and the level with the greatest bilateral mean transverse process length were identified using both morphological and presacral numbering. Bilateral asymmetry at each morphological level was defined as the absolute difference between the corresponding left- and right-sided transverse process lengths.

Additionally, the position of the iliac crest line was recorded in relation to the vertebral levels in both morphological (L1–L5) and presacral numbering systems. Both predefined binary landmarks and the exact vertebral level of the iliac crest were retained for analysis. All measurements and level assignments were documented in a dedicated spreadsheet for subsequent analysis.

### 2.6. Statistical Analysis

Categorical variables were expressed as counts and percentages. For proportions presented for individual segmentation groups, 95% confidence intervals were calculated using the Wilson score method. Associations between thoracic segmentation patterns and binary anatomical markers, including transverse process-based and iliac crest-based markers, were assessed using the two-sided Fisher–Freeman–Halton exact test for 3 × 2 contingency tables. For contingency tables with more than two outcome categories, *p*-values were estimated using Monte Carlo simulation with 1,000,000 random tables generated under fixed marginal totals, using the Pearson chi-square statistic. A fixed random seed (20261004) was used to ensure reproducibility, and Monte Carlo *p*-values were calculated using the +1 correction. Effect sizes were quantified using Cramér’s V derived from Pearson’s chi-square statistic. Secondary analyses were performed according to lumbar vertebral count, and a sensitivity analysis compared standard C7T12L5 cases with non-standard configurations. Multiple testing across the nine primary anatomical-marker analyses was addressed using the Holm procedure. Secondary and sensitivity analyses were considered exploratory and were not included in the Holm correction family. Continuous transverse process measurements were summarized as mean ± standard deviation and range. All tests were two-sided, and a Holm-adjusted *p*-value < 0.05 for the primary analyses was considered statistically significant. Descriptive statistics were calculated using STATISTICA version 13.3 (TIBCO Software, Palo Alto, CA, USA). Exact and Monte Carlo analyses of contingency tables were performed using Python version 3.13.5 with SciPy version 1.17.0. Inter-observer reliability of transverse process measurements was assessed using a two-way mixed-effects intraclass correlation coefficient for absolute agreement and single measurements, with 95% confidence intervals.

### 2.7. STROBE Statement

This retrospective observational study was reported in accordance with the Strengthening the Reporting of Observational Studies in Epidemiology (STROBE) guidelines [23]. The completed STROBE checklist is provided as Supplementary Material.

## 3. Results

A total of 66 cases were included in the analysis. The study group comprised 46 men (69.7%) and 20 women (30.3%), with a mean age of 42.6 ± 19.9 years (median 36.5, range 19–91). Thoracic segmentation patterns were distributed as follows: C7T11 in 10 cases (15.2%), C7T12 in 43 (65.2%), and C7T13 in 13 (19.7%). Within the C7T12 group, lumbar segmentation comprised C7T12L4 in 8 cases, C7T12L5 in 17, and C7T12L6 in 18.

Within the selected study cohort, using morphological lumbar numbering, the longest transverse process was most commonly identified at L3 (45/66, 68.2%), followed by L4 (14/66, 21.2%) and L2 (7/66, 10.6%). Based on the bilateral mean length, L3 was again the most frequent level (49/66, 74.2%), followed by L4 (13/66, 19.7%) and L2 (4/66, 6.1%). Using presacral numbering, the longest transverse process was most frequently located at vertebra 22 (44/66, 66.7%), as was the longest bilateral pair (46/66, 69.7%).

**Figure 3:**
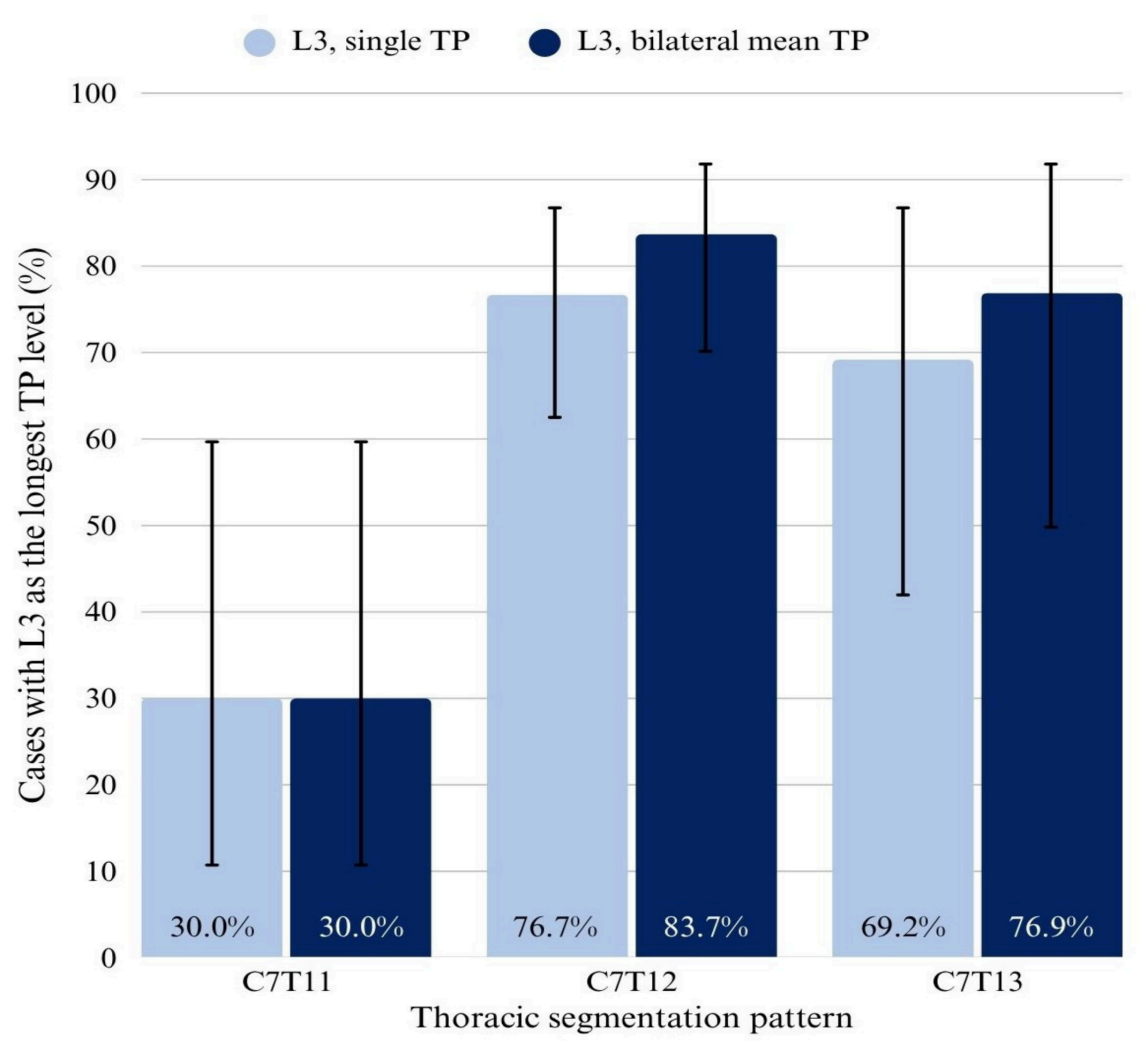
Proportion of cases with L3 identified as the longest transverse process level according to thoracic segmentation pattern, based on the single longest process and bilateral mean transverse process length. Error bars indicate 95% Wilson confidence intervals. TP, transverse process.

The distribution of the proposed transverse process-based markers varied across thoracic segmentation patterns. The proportion of cases in which L3 was identified as the longest transverse process level differed across C7T11, C7T12, and C7T13 configurations, both for the single longest transverse process (30.0%, 76.7%, and 69.2%, respectively; exact *p* = 0.019, Cramér’s V = 0.35) and for the bilateral mean length (30.0%, 83.7%, and 76.9%, respectively; exact *p* = 0.003, Cramér’s V = 0.43). After correction for multiple testing, the association based on the bilateral mean remained significant (Holm-adjusted *p* = 0.014), whereas the single-process analysis did not (Holm-adjusted *p* = 0.058) (Table 1; Figure 3).

**Figure 4:**
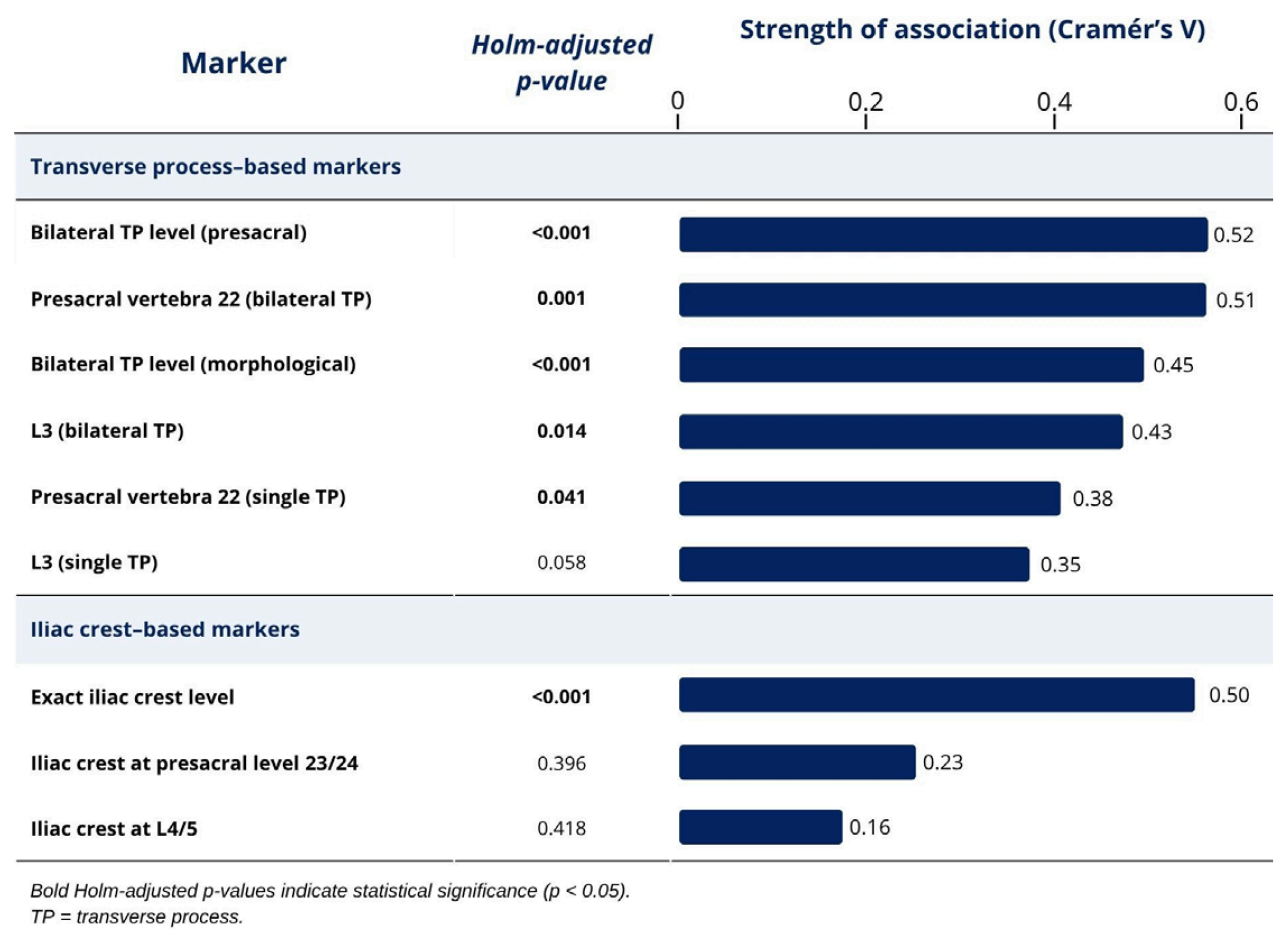
Strength of association between proposed anatomical markers and thoracic segmentation patterns, expressed as Cramér’s V. Holm-adjusted *p*-values are shown, with statistically significant adjusted *p*-values presented in bold. TP, transverse process.

Similarly, the proportion of cases in which the 22nd presacral vertebra was identified as the level bearing the longest transverse process differed across segmentation patterns, both for the single longest transverse process (70.0%, 76.7%, and 30.8%, respectively; exact *p* = 0.010, Cramér’s V = 0.38) and for the bilateral mean length (70.0%, 83.7%, and 23.1%, respectively; exact *p* < 0.001, Cramér’s V = 0.51). Both associations remained significant after Holm correction (adjusted *p* = 0.041 and 0.001, respectively) (Table 1; Figure 4).

Analysis of the exact level of the longest bilateral pair further demonstrated differences across thoracic segmentation patterns. Using morphological numbering, the longest pair was most commonly located at L4 in C7T11 (70.0%), whereas L3 predominated in C7T12 (83.7%) and C7T13 (76.9%). The distribution of the longest level differed significantly across thoracic segmentation patterns (Monte Carlo *p* < 0.001, Cramér’s V = 0.45). Using presacral numbering, vertebra 22 predominated in C7T11 (70.0%) and C7T12 (83.7%), whereas the maximum shifted to vertebra 23 in C7T13 (76.9%), with a larger effect size for the association with thoracic segmentation (Monte Carlo *p* < 0.001, Cramér’s V = 0.52). Both associations remained significant after correction for multiple testing (Holm-adjusted *p* < 0.001) (Figure 4).

In contrast, neither of the binary iliac crest-based markers was significantly associated with thoracic segmentation. The proportion of cases with the iliac crest at the 23/24 presacral level was observed in 20.0%, 51.2%, and 53.8% of C7T11, C7T12, and C7T13 cases, respectively (exact *p* = 0.198, Cramér’s V = 0.23). Similarly, the proportion of cases with the iliac crest at L4/5 did not differ significantly across segmentation patterns (50.0%, 51.2%, and 30.8%, respectively; exact *p* = 0.418, Cramér’s V = 0.16). However, when the exact vertebral level of the iliac crest was considered, its distribution differed significantly according to thoracic segmentation. The iliac crest was most commonly located at L5 in C7T11 (80.0%) and at L4 in C7T13 (92.3%) (Monte Carlo *p* < 0.001, Cramér’s V = 0.50). The association remained significant after correction for multiple testing (Holm-adjusted *p* < 0.001) (Figure 4).

In secondary analyses according to lumbar vertebral count, 11 patients had four lumbar vertebrae, 31 had five, and 24 had six. L3 was the level bearing the single longest transverse process in 7/11 (63.6%), 26/31 (83.9%), and 12/24 (50.0%) of these groups, respectively (exact *p* = 0.021, Cramér’s V = 0.33). Based on bilateral mean length, the corresponding proportions were 8/11 (72.7%), 27/31 (87.1%), and 14/24 (58.3%), respectively (exact *p* = 0.042, Cramér’s V = 0.30).

In the sensitivity analysis, L3 was the level bearing the single longest transverse process in 15/17 (88.2%) patients with standard C7T12L5 segmentation and in 30/49 (61.2%) patients with non-standard configurations (Fisher’s exact *p* = 0.067). Based on bilateral mean length, the corresponding proportions were 15/17 (88.2%) and 34/49 (69.4%), respectively (*p* = 0.199).

Absolute transverse process measurements are summarized in Table 2. Mean left- and right-sided TP lengths and bilateral side-to-side differences are reported for morphological levels L1–L5 according to thoracic segmentation pattern.

In the inter-observer reliability analysis, all 66 studies were independently re-measured by a neurosurgeon who had no access to the original measurements. Agreement with the original measurements was good to excellent: the intraclass correlation coefficient (ICC, two-way mixed-effects model, absolute agreement, single measures) was 0.88 (95% CI: 0.85–0.91) for individual left- and right-sided transverse-process lengths and 0.93 (95% CI: 0.90–0.94) for bilateral mean lengths. Intra-observer reliability was not assessed.

## 4. Discussion

Accurate vertebral enumeration remains challenging when imaging is restricted to the lumbosacral region, particularly in the presence of numerical or transitional vertebral variants [1,2,5,6,8]. Although several anatomical landmarks have been proposed to assist numbering on limited-field imaging, none consistently reproduces whole-spine enumeration across different segmentation patterns [1,5,6,14,15,16,17,18].

Although numbering discrepancies may have limited immediate consequences in some non-interventional settings, precise identification is critical in interventional procedures and spinal surgery, where incorrect numbering may contribute to wrong-level interventions and associated clinical and medicolegal consequences [1,2,13].

In most patients included in the present study, the longest lumbar transverse process was located at L3, which was recently proposed as a potential landmark [19]. However, the frequency of this finding varied according to the number of thoracic vertebrae: L3 was the level bearing the longest transverse process in only 30.0% of C7T11 cases, compared with 76.7% of C7T12 and 69.2% of C7T13 cases when the single longest process was considered. Using bilateral mean length, the corresponding proportions were 30.0%, 83.7%, and 76.9%, respectively. Presacral numbering did not resolve this limitation. Although the 22nd presacral vertebra most frequently bore the longest transverse process in C7T11 and C7T12, the maximum shifted predominantly to vertebra 23 in C7T13. This indicates that the presacral identity of the longest transverse process also changes with thoracic segmentation. Consequently, L3 as the level bearing the longest transverse process cannot be considered a segmentation-independent marker of lumbar vertebral identity.

These contrasts should be read with their precision. The C7T11 estimate of 30% had a Wilson interval of 10.8–60.3% and overlapped the C7T13 interval. In C7T11, mean L3 and L4 lengths were almost equal (left, 24.7 mm and 25.0 mm), whereas in C7T12 L3 was clearly longer (27.6 mm versus 24.4 mm). The near-equivalence of L3 and L4 in C7T11 may reflect altered thoracolumbar regional patterning associated with transitional anatomy [20,24,25], although the underlying developmental mechanism cannot be determined from the present data. Secondary stratification by lumbar count also shifted the landmark: L3 was the single longest process in 83.9% of five-lumbar spines but only 50.0% of six-lumbar spines. In the sensitivity analysis, L3 was longest in 15/17 (88.2%) standard C7T12L5 spines and 30/49 (61.2%) non-standard configurations (*p* = 0.067; bilateral means, 88.2% versus 69.4%, *p* = 0.199). The high proportion of L3-dominant cases observed in the standard C7T12L5 subgroup is broadly consistent with the original observation by Rädel and Paton [19]. However, the lower proportions observed in non-standard configurations suggest that the apparent reliability of this landmark depends on vertebral segmentation rather than representing a fixed anatomical relationship. The thoracic association in the primary analysis is therefore not cleanly separable from lumbar transitional anatomy, and the reference comparison is underpowered.

The two binary iliac crest-based markers were not significantly associated with thoracic segmentation. However, when iliac crest position was assessed at the exact vertebral level, a strong association with thoracic segmentation was observed, with the crest most commonly located at L5 in C7T11 and at L4 in C7T13. The lack of significant associations for the binary definitions should not be interpreted as evidence of segmentation independence, particularly given the limited subgroup sizes. These findings further illustrate the difficulty of deriving a universally applicable vertebral landmark from a single anatomical relationship on limited-field imaging [5,6,16,17,18].

Recent studies have shifted focus from isolated anatomical landmarks toward more comprehensive morphological assessment of the lumbosacral and thoracolumbar junctions [24,25,26,27]. Krenn et al. reported that segmentation anomalies of the sacrum are frequent (approximately 40% prevalence) and produce predictable morphological patterns that can help distinguish lumbarization from sacralization [28]. Similarly, Wu et al. recently proposed a rapid, practical method for identifying lumbarization on standard lumbar radiographs and CT based on specific sacral and L5 morphological features [29]. These morphology-based approaches provide an alternative to single-landmark methods by incorporating the broader morphology of transitional regions [24,25,26,27,28,29].

## 5. Limitations

First, although the overall cohort allowed assessment of the proposed anatomical relationships, the subgroups with non-standard thoracic segmentation (C7T11 and C7T13) were relatively small, which may have limited the precision of some estimates and resulted in sparse contingency tables requiring exact statistical methods. The C7T12L5 reference group comprised the 17 examinations that remained after sequential exclusion of cases meeting Jenkins or Castellvi criteria for lumbosacral transitional anatomy, followed by exclusion of cervical, thoracolumbar, and sacral anomalies. It is therefore a highly selected anatomical reference set. Comparisons with this group have limited precision, and cohort percentages should not be interpreted as population prevalences. Second, inter-observer agreement was assessed by independent re-measurement of all 66 studies, but intra-observer reliability was not evaluated. Repeated measurements by the same reader might have quantified within-reader variability in localizing the pedicle–lamina junction and the transverse-process tip. Third, the retrospective design and single-center setting limit the generalizability of our findings to other populations and imaging protocols.

Fourth, the study was conducted using trauma CT examinations; therefore, the findings may not be directly generalizable to patients undergoing elective spine imaging, in whom degenerative changes or differences in positioning could influence the appearance and identification of the evaluated anatomical landmarks. Finally, the study did not directly compare the diagnostic performance of transverse process-based markers with alternative morphology-based approaches within the same cohort.

## 6. Conclusions

While a simple, reproducible bony landmark on routine lumbosacral imaging remains attractive, single-parameter approaches based on L3 transverse process length are limited by variation in thoracic vertebral count. Morphology-based approaches evaluating transitional regions may offer a more comprehensive alternative to isolated anatomical landmarks, although their comparative performance requires direct validation [24,25,26,27,28,29]. Until a reliable method for vertebral segmentation on limited imaging is established, whole-spine counting should remain the reference whenever precise vertebral numbering is clinically required [1,2,3,4].

## Figures and Tables

**Table 1:** Associations between proposed anatomical markers and thoracic segmentation patterns.

Marker	C7T11 (*n* = 10)	C7T12 (*n* = 43)	C7T13 (*n* = 13)	Raw *p*-Value	Holm-Adjusted *p*-Value	Cramér’s V
L3 longest (single)	30.0% (10.8–60.3)	76.7% (62.3–86.8)	69.2% (42.4–87.3)	0.019	0.058	0.35
L3 longest (bilateral mean)	30.0% (10.8–60.3)	83.7% (70.0–91.9)	76.9% (49.7–91.8)	0.003	**0.014**	0.43
22nd vertebra longest (single)	70.0% (39.7–89.2)	76.7% (62.3–86.8)	30.8% (12.7–57.6)	0.010	**0.041**	0.38
22nd vertebra longest (bilateral mean)	70.0% (39.7–89.2)	83.7% (70.0–91.9)	23.1% (8.2–50.3)	<0.001	**0.001**	0.51
Iliac crest at 23/24 presacral	20.0% (5.7–51.0)	51.2% (36.8–65.4)	53.8% (29.1–76.8)	0.198	0.396	0.23
Iliac crest at L4/L5 level	50.0% (23.7–76.3)	51.2% (36.8–65.4)	30.8% (12.7–57.6)	0.418	0.418	0.16

Values are presented as percentages with 95% Wilson confidence intervals in parentheses. Associations were assessed using the two-sided Fisher–Freeman–Halton exact test. Holm adjustment was applied across nine primary analyses, including three exact-level analyses presented separately in Figure 4. Significant Holm-adjusted *p*-values are shown in bold. Effect sizes are reported as Cramér’s V. The 22nd vertebra refers to presacral numbering.

**Table 2:** Absolute lumbar transverse process lengths and bilateral asymmetry according to thoracic segmentation pattern.

Thoracic Patterns	Level	Left TP Length, mm	Right TP Length, mm	Bilateral Asymmetry, mm
**C7T11**	**L1**	17.3 ± 4.1 (10.8–24.9)	16.9 ± 4.6 (11.4–28.5)	4.7 ± 3.4 (1.3–10.4)
	**L2**	21.6 ± 2.1 (18.0–24.8)	21.4 ± 3.4 (17.0–24.9)	1.8 ± 1.3 (0.0–4.5)
	**L3**	24.7 ± 3.8 (20.0–30.5)	25.4 ± 3.6 (21.2–31.5)	2.3 ± 2.0 (0.3–6.7)
	**L4**	25.0 ± 2.4 (21.2–28.1)	24.5 ± 1.9 (22.3–27.8)	1.7 ± 1.2 (0.2–3.8)
	**L5**	19.8 ± 2.1 (16.3–23.8)	19.1 ± 2.6 (13.9–21.6)	1.5 ± 1.0 (0.0–2.6)
**C7T12**	**L1**	19.2 ± 4.4 (4.0–26.8)	19.1 ± 4.0 (10.2–29.3)	2.4 ± 1.8 (0.1–7.5)
	**L2**	23.7 ± 3.7 (16.0–31.7)	23.8 ± 4.1 (14.4–33.6)	1.9 ± 1.5 (0.0–5.6)
	**L3**	27.6 ± 3.8 (17.4–34.8)	27.5 ± 4.0 (17.6–35.8)	2.0 ± 1.5 (0.0–7.6)
	**L4**	24.4 ± 3.8 (17.5–35.8)	23.2 ± 4.2 (15.0–35.9)	2.5 ± 2.2 (0.1–8.6)
	**L5**	21.4 ± 3.8 (13.3–33.8)	20.3 ± 3.7 (12.9–28.9)	3.3 ± 2.5 (0.0–9.9)
**C7T13**	**L1**	22.2 ± 3.5 (17.8–29.9)	21.2 ± 3.0 (16.1–27.3)	2.4 ± 1.5 (0.2–5.9)
	**L2**	27.5 ± 4.4 (21.5–36.7)	25.9 ± 2.7 (21.0–32.3)	2.0 ± 2.8 (0.3–10.0)
	**L3**	27.9 ± 4.1 (19.9–33.4)	27.0 ± 3.2 (22.2–33.6)	2.0 ± 1.7 (0.3–6.5)
	**L4**	22.9 ± 3.7 (16.7–32.1)	22.1 ± 4.0 (13.9–29.7)	2.3 ± 2.0 (0.5–8.1)
	**L5**	19.5 ± 3.3 (15.1–24.6)	20.8 ± 4.8 (15.9–32.4)	2.5 ± 3.2 (0.3–11.2)

Values are presented as mean ± standard deviation (range). Bilateral asymmetry was defined as the absolute difference between left- and right-sided transverse process lengths at the same morphological level. In C7T12, one right-sided measurement was unavailable at L1 and L4; for L5, *n* = 35. For C7T13 L5, *n* = 10 because this morphological level was absent in C7T13L4 configurations. TP, transverse process.

## Data Availability

The data presented in this study are available on request from the corresponding author due to ethical and privacy restrictions.

## Supplementary Materials

The following supporting information can be downloaded at https://www.mdpi.com/article/10.3390/diagnostics16203409/s1, STROBE checklist. Reference [30] is cited in Supplementary Materials.

## Abbreviations

The following abbreviations are used in this manuscript:

AI	artificial intelligence
CT	computed tomography
LSTV	lumbosacral transitional vertebrae
MPR	multiplanar reconstruction
