# Supplementary material for: Reliability of the L3 Transverse Process as a Landmark for Lumbar Spine Enumeration Across Different Vertebral Segmentation Patterns: A Retrospective CT Study
Source: Diagnostics (Basel). 2026 Oct 20;16(20):3409. doi: 10.3390/diagnostics16203409 (PMC13703058; doi:10.3390/diagnostics16203409)
Supplement: Supplementary file 1 [file diagnostics-16-03409-s001.zip › diagnostics-4522874-supplementary.pdf]

STROBE checklist

Strengthening the Reporting of Observational Studies in Epidemiology — checklist for cross-sectional studies

Manuscript. Reliability of the L3 Transverse Process as a Landmark for Lumbar Spine Enumeration across Different Vertebral Segmentation Patterns: A Retrospective CT Study

Authors. Jakub Jankowski, Natalia Maślanek, Marcin Glin, Marcin Jasiewicz, Agnieszka Pawełczyk, Michał Polguj, Maciej Radek

Study design. Retrospective cross-sectional observational study of whole-spine trauma CT examinations (enriched for non-standard thoracolumbar formulas).

Checklist used. STROBE statement, cross-sectional version. Cohort-only items (matching, follow-up, outcome events over time) do not apply and are not listed. Locations refer to manuscript sections because the submission file is not yet paginated.

Item	Recommendation	Reported in the manuscript
**Title and abstract**		
1a	Indicate the study's design with a commonly used term in the title or the abstract.<br>*Design is named as a retrospective CT study. The enriched, non-probability construction of the sample is stated in the abstract.*	**Title; Abstract (Methods)**
1b	Provide in the abstract an informative and balanced summary of what was done and what was found.<br>*Results give counts, proportions, exact and Holm-adjusted p values, and explicitly state that the percentages are not population prevalences.*	**Abstract (Background/Objectives, Methods, Results, Conclusions)**
**Introduction**		
2	Explain the scientific background and rationale for the investigation being reported.<br>*Restricted-field numbering, LSTV and numerical variants, limitations of existing landmarks, and the Rädel and Paton transverse-process proposal.*	**Section 1, Introduction**
3	State specific objectives, including any prespecified hypotheses.<br>*Objective stated: whether L3 as the level with the longest transverse process is independent of thoracic and lumbar count. No separately worded prespecified statistical hypothesis.*	**Section 1, final paragraph; Abstract (Background/Objectives)**
**Methods**		
4	Present key elements of study design early in the paper.<br>*Retrospective observational study; ethics approval RNN/09/25/KE; consent waived for anonymized CT data.*	**Section 2.1**
5	Describe the setting, locations, and relevant dates, including periods of recruitment, exposure, follow-up, and data collection.<br>*Emergency department of a tertiary referral hospital in Poland; whole-body trauma CT, January 2022–December 2024. No longitudinal follow-up (cross-sectional imaging review).*	**Section 2.2**
6	Give the eligibility criteria, and the sources and methods of selection of participants.<br>*Source: 499 trauma CT examinations with confirmed numbering. All 49 non-standard thoracolumbar formulas included. Reference arm: 17 C7T12L5 spines remaining after Jenkins, Castellvi, cervical, thoracolumbar, and sacral filters. Most recent scan if repeated. Exclusions: incomplete thoracolumbar visualization or image quality precluding measurement.*	**Sections 2.2 and 2.4; Figure 1**
7	Clearly define all outcomes, exposures, predictors, potential confounders, and effect modifiers. Give diagnostic criteria, if applicable.<br>*Grouping variable: thoracic pattern (C7T11, C7T12, C7T13). Secondary grouping: lumbar count (L4, L5, L6). Outcomes: level of the single longest transverse process and of the greatest bilateral mean, in morphological and presacral numbering; binary and exact iliac-crest landmarks. Castellvi and Jenkins used as exclusion filters, not as study outcomes.*	**Sections 2.4 and 2.5**
8*	For each variable of interest, give sources of data and details of methods of assessment (measurement). Describe comparability of assessment methods if there is more than one group.<br>*Optima 540, 120 kV, 1.25 mm reconstructions; RadiAnt MPR and 3D. Length from pedicle–lamina junction to tip, after Rädel et al. Same protocol in all groups. Senior consensus review; independent re-measurement of all 66 studies.*	**Sections 2.2, 2.3 and 2.5; Figure 2**
9	Describe any efforts to address potential sources of bias.<br>*Whole-spine counting as the numbering reference; consensus adjudication; blinded re-measurement; complete inclusion of non-standard formulas; Holm correction of nine primary tests; enrichment and selection of the reference arm stated as limitations.*	**Sections 2.3, 2.4 and 2.6; Section 5**

Item	Recommendation	Reported in the manuscript
**10**	Explain how the study size was arrived at.<br>*No a priori sample-size calculation. Size is the complete set of non-standard formulas in the source series plus every C7T12L5 spine remaining after the stated filters (n = 66).*	**Section 2.4; Figure 1**
**11**	Explain how quantitative variables were handled in the analyses. If applicable, describe which groupings were chosen, and why.<br>*Lengths summarized as mean ± SD and range. Binary markers and exact-level distributions analysed separately. Primary grouping by rib-bearing thoracic count; secondary grouping by lumbar count; sensitivity contrast of standard C7T12L5 versus all non-standard configurations.*	**Sections 2.4, 2.5 and 2.6**
**12a**	Describe all statistical methods, including those used to control for confounding.<br>*Wilson score intervals; Fisher–Freeman–Halton exact test; Monte Carlo Pearson tests (1,000,000 tables, seed 20261004, +1 correction); Cramér's V; Holm adjustment across nine primary analyses. No regression adjustment for confounding.*	**Section 2.6**
**12b**	Describe any methods used to examine subgroups and interactions.<br>*Secondary stratification by lumbar vertebral count. Individual thoracolumbar formulas retained descriptively. No formal interaction model.*	**Sections 2.4 and 2.6; Results**
**12c**	Explain how missing data were addressed.<br>*Partial measurements reported with reduced denominators (C7T12: one right-sided value unavailable at L1 and L4; L5, n = 35; C7T13 L5, n = 10 because the level is absent in C7T13L4). No imputation.*	**Table 2, footnote**
**12d**	If applicable, describe analytical methods taking account of sampling strategy.<br>*Not a complex survey sample. Consecutive institutional series with complete inclusion of non-standard formulas; enrichment is described and percentages are not treated as population prevalences.*	**Sections 2.4 and 2.6; Abstract**
**12e**	Describe any sensitivity analyses.<br>*Standard C7T12L5 (n = 17) versus pooled non-standard configurations (n = 49), for the single longest process and the bilateral mean. Exploratory; outside the Holm family.*	**Sections 2.4 and 2.6; Results**
**Results**		
**13a***	Report numbers of individuals at each stage of study—e.g. numbers potentially eligible, examined for eligibility, confirmed eligible, included in the study, and analysed.<br>*499 numbered; 49 non-standard included in full; 450 C7T12L5 reduced by successive filters to 37, 33, 31, 29, and 17; analysed n = 66.*	**Section 2.4; Section 3, opening paragraph; Figure 1**
**13b***	Give reasons for non-participation at each stage.<br>*Incomplete thoracolumbar visualization or non-measurable image quality; sequential exclusion of Jenkins-positive, Castellvi-positive, cervical, thoracolumbar, and sacral anomalies from the reference arm only.*	**Sections 2.2 and 2.4; Figure 1**
**13c***	Consider use of a flow diagram.<br>*Flow diagram of selection and allocation to the final groups.*	**Figure 1**
**14a***	Give characteristics of study participants (e.g. demographic, clinical, social) and information on exposures and potential confounders.<br>*46 men (69.7%) and 20 women (30.3%); age 42.6 ± 19.9 years (median 36.5, range 19–91). Thoracic and lumbar formulas reported. No clinical outcome covariates.*	**Section 3, first paragraph**
**14b***	Indicate the number of participants with missing data for each variable of interest.<br>*Missing or structurally absent transverse-process measurements are denominated in the table footnote. No separate missing-data table.*	**Table 2, footnote**
**15***	Report numbers of outcome events or summary measures.<br>*Level of the longest process and of the longest bilateral pair, overall and by thoracic pattern; absolute lengths and asymmetry.*	**Section 3; Tables 1 and 2; Figures 3 and 4**
**16a**	Give unadjusted estimates and, if applicable, confounder-adjusted estimates and their precision (e.g. 95% confidence interval). Make clear which confounders were adjusted for and why they were included.<br>*Group percentages with 95% Wilson intervals, exact p values, Holm-adjusted p values, and Cramér's V. Estimates are unadjusted. No confounder-adjusted model.*	**Table 1; Section 3**
**16b**	Report category boundaries when continuous variables were categorized.<br>*Thoracic categories are rib counts (11, 12, 13). Lumbar categories are vertebral counts (4, 5, 6). Iliac-crest binaries are prespecified (23/24 presacral; L4/L5); exact level is also reported.*	**Sections 2.4 and 2.5; Table 1**

Item	Recommendation	Reported in the manuscript
**16c**	If relevant, consider translating estimates of relative risk into absolute risk for a meaningful time period.<br>*Cross-sectional anatomical association. No time-to-event or period risk.*	**Not applicable**
**17**	Report other analyses done—e.g. analyses of subgroups and interactions, and sensitivity analyses.<br>*Lumbar-count stratification; standard versus non-standard comparison; ICC 0.88 (0.85–0.91) for single lengths and 0.93 (0.90–0.94) for bilateral means.*	**Section 3, secondary and sensitivity paragraphs; inter-observer paragraph**
**Discussion**		
**18**	Summarise key results with reference to study objectives.<br>*L3 and the 22nd presacral vertebra both vary with thoracic pattern; neither is treated as segmentation-independent.*	**Section 4, paragraphs 3–5; Section 6**
**19**	Discuss limitations of the study, taking into account sources of potential bias or imprecision. Discuss both direction and magnitude of any potential bias.<br>*Small C7T11 and C7T13 groups and Wilson-interval overlap; selected reference arm; no intra-observer reliability; single centre; trauma rather than elective imaging; no head-to-head comparison with morphology-based methods. Enrichment precludes prevalence interpretation.*	**Section 5; Section 4, paragraph 4**
**20**	Give a cautious overall interpretation of results considering objectives, limitations, multiplicity of analyses, results from similar studies, and other relevant evidence.<br>*Primary multiplicity handled with Holm. Agreement with Rädel and Paton confined to the standard C7T12L5 subset. Morphology-based alternatives cited but not tested in this cohort.*	**Sections 4 and 6**
**21**	Discuss the generalisability (external validity) of the study results.<br>*Single-centre trauma CT; may not extend to elective imaging, other protocols, or populations with different degenerative or positional characteristics.*	**Section 5, third and fourth limitations**
**Other information**		
**22**	Give the source of funding and the role of the funders for the present study and, if applicable, for the original study on which the present article is based.<br>*No external funding. Ethics: Bioethics Committee of the Medical University of Lodz, RNN/09/25/KE. Informed consent waived.*	**Funding; Institutional Review Board Statement**

Notes on completion

Items marked with an asterisk (*) are those for which the STROBE Explanation and Elaboration article asks authors to give information separately for exposed and unexposed groups, where applicable. Here the groups are thoracic segmentation patterns, and the corresponding stratified results are in Table 1, Table 2, and Figures 3 and 4.

Item 10 is reported as a complete enumeration of eligible examinations, not as a calculated sample size. Item 12a describes exact tests and multiplicity control, not confounder-adjusted regression. Item 16c is not applicable. These entries are intentional and match the analysis actually performed.

The manuscript states that the study was reported in accordance with STROBE (Section 2.7). This file is the supplementary checklist referred to there.

Reference

von Elm E, Altman DG, Egger M, Pocock SJ, Gøtzsche PC, Vandenbroucke JP; STROBE Initiative. The Strengthening the Reporting of Observational Studies in Epidemiology (STROBE) statement: guidelines for reporting observational studies. Ann Intern Med. 2007;147:573–577. doi:10.7326/0003-4819-147-8-200710160-00010.

Vandenbroucke JP, von Elm E, Altman DG, et al. Strengthening the Reporting of Observational Studies in Epidemiology (STROBE): explanation and elaboration. Ann Intern Med. 2007;147:W163–W194. doi:10.7326/0003-4819-147-8-200710160-00010-w1.

Checklist wording follows the STROBE cross-sectional checklist (STROBE Initiative, strobe-statement.org). This document is supplementary material for the manuscript named above and is not a substitute for the Explanation and Elaboration article.
